# Association of alcohol consumption and components of metabolic syndrome among people in rural China

**DOI:** 10.1186/s12986-015-0007-4

**Published:** 2015-02-28

**Authors:** Jing Xiao, Jian-Ping Huang, Guang-Fei Xu, De-Xi Chen, Gui-Yun Wu, Min Zhang, Yi Shen, Hui Cai

**Affiliations:** Department of Epidemiology and Medical Statistics, School of Public Health, Nantong University, Nantong, Jiangsu China; Department of Chronic Disease and Prevention Center, Center for Disease Control and Prevention of Nantong, Nantong, Jiangsu China; Department of Nutrition and Food Science, School of Public Health, Nantong University, Nantong, Jiangsu China

**Keywords:** Metabolic syndrome, Components, Alcohol consumption, Rice wine, Liquor

## Abstract

**Background:**

Accumulative evidence in the literature suggests alcohol consumption is a protective factor of the metabolic syndrome (MS). However, few studies investigated the relationship between alcohol consumption and components of MS. We examined association of several types of alcoholic beverage with components of MS among people in rural China.

**Methods:**

In the Nantong Metabolic Syndrome Study (NMSS), a cross-sectional study, a total of 20,502 participants, including 13,505 women and 6,997 men aged 18–74 years, were recruited between 2007 and 2008 in Nantong, China. Socio-economic status, dietary intake, physical exercise, alcoholic beverage consumption, and smoking status information were obtained, and triglycerides (TG), high-density lipoprtein cholesterol (HDL-c), blood pressure (BP) and blood glucose level were examined for all participants. Logistic regression model and the restricted cubic spline approach were used to analyze the associations between alcoholic beverage consumption and MS components.

**Results:**

The MS prevalence was 21.1% in the whole population, which was significantly low among drinkers (20.6%), compared with non-drinkers (23.6%) in women, and was comparable in men (16.4% versus 17.1%). High HDL-c level was observed among drinkers, compared with non-drinkers in both men and women. Low TG level and Systolic BP (SBP) were found only among rice wine drinkers in women, and high waist circumference, high TG and BP were found among beer and liquor drinkers in men. Furthermore, we found that the highest quartile of rice wine drink in women may decrease 24% risk of high TG, 30% risk of low HDL-c and 43% risk of high glucose among MS components cases respectively, compared with non-drinkers (p for trend <0.01 for those three components). While compared non-drinkers among men, the highest quartile of liquor drink may increase 32% risk of high SBP, 55% risk of high Diastolic BP (DBP) and 34% risk of abdominal obesity among MS components cases respectively, but decrease 45% risk of low HDL-c (p for trend <0.05 for those four components).

**Conclusion:**

Our data suggested that all alcoholic beverages increased HDL-c level. Rice wine decreased both TG level and blood glucose in women only and it could be one of healthy alcoholic beverages in MS prevention in Chinese women. While excessive liquor consumption increased BP and waist circumference level and it may lead to hypertension and central obesity in Chinese men.

## Introduction

Metabolic syndrome (MS) is a constellation of metabolic abnormalities, including abdominal obesity, hypertriglyceridemia, low high-density lipoprotein (HDL) concentrations, hypertension, and hyperglycaemia [1], which is strong association with the development of type II diabetes and risk of cardiovascular morbidity and mortality [2,3]. Ford *et al*. reported that according to International Diabetes Federation (IDF) criteria the prevalence of MS is 23% in America, and 23.50% and 14.70% in urban and rural areas in China, respectively [4]. Prevalence and incidence of MS increase rapidly and become a major public health challenge worldwide [5]. The etiology of the metabolic syndrome is complex, determined by the interplay of both genetic and environmental factors [6]. There is increasing interest to obtain a greater understanding of the modifiable factors that may mitigate or moderate the progression of MS leading towards its development.

Alcohol consumption is one of most prevalent lifestyles in the world [7]. It has recently been interested in its association with risk of MS and its components. Several studies reported that alcohol consumption has a higher risk of developing type II diabetes [8,9], hypertension [8,10-12], obesity [8,13-15], high triglycerides (TG) [10,16,17] and high fasting glucose [1], which are the principal components of MS [8]. Sayon *et al*. found that severe drinkers were associated with weight gain while light to moderate alcohol drinkers, especially wine drinkers, was not associated with obesity [18]. However, many studies have suggested that alcohol consumption is a protective effect on MS prevalence and incidence by increasing HDL cholesterol (HDL-c) [10,19,20]. Stoutenberg *et al*. reported that all levels of alcohol consumption provided significant inverse associations with MS but alcohol consumption was not significantly associated with central obesity, hypertriacylglycerolemia or hypertension [21]. Several studies have further reported that moderate alcohol consumption was associated with a lower risk of diabetes [22-24]. Take these inconsistent results into consideration, it is unclear about association between different alcoholic beverages and MS components. Recently, several studies conducted in China have evaluated association between alcohol consumption and MS among Chinese minors [11] and Chinese men [1]. Few studies focus on evaluating the associations between the amount and types of alcoholic beverage consumed and MS components among Chinese adults.

We launched the Nantong Metabolic Syndrome Study (NMSS) in 2007. The NMSS is a population-based cross-sectional study of 20,502 participants (6,997 men and 13,505 women) aged 18–74 years in the rural areas of Nantong, China. In current study we will evaluate association between several alcoholic beverages and MS components in both men and women living in rural China and to our knowledge, this is the largest study on association between alcohol consumption and each of MS components in rural China.

## Methods

### Study population

The NMSS recruitment began in July 2007 and was completed in August 2008. The details of the NMSS have been described elsewhere [25]. Briefly, we recruited 24,519 residents between the ages of 18 and 74 years from two townships in rural Nantong. Among them, 20,502 participants, including 6,997 men and 13,505 women, were enrolled in the study, with a response rate of 83.6%. The reasons for non-participation were refusal (3.21%), out of the area during enrollment (7.21%), and other miscellaneous reasons, such as poor health or hearing problems (5.98%). In our study, a rural area was defined as an area with a primary administrative unit termed a ‘village’. At the time of interview, most participants (99.5%) lived in a village, and 13,306 (64.9%) people were farmers. The study protocols were approved by the Boards of Scientific Research of Nantong University and the Nantong Centers for Disease Control (CDC), and all participants provided written informed consent. Socio-economic factors, dietary intake, physical activity, alcohol consumption, smoking status, personal medical history and family history of several chronic diseases were assessed using the same standard questionnaires. The height, weight, and waist circumference of each participant was measured according to standard protocols.

The participants were asked the age they started and stopped smoking and how many cigarettes they consumed per day. We defined ever smokers as participants who had smoked at least 100 cigarettes in their lifetimes. Each participant were asked about monthly alcohol consumption within the recent year, they provided the usual alcohol amount of grape wine, rice wine, beer and liquor consumed on a monthly basis separately. One drink was defined as 4 ounces of grape wine, 4.8 ounces of rice wine, a 12 ounce of beer or 1 ounce of liquor, all of which contain approximately 0.5 ounces of absolute alcohol [26]. A total of 25.6% (11.2% female and 53.6% male) of the participants in our study consumed alcohol, 66.2% (66.6% male and 65.1% female respectively) of whom reportedly consumed rice wine; 1.8% was grape wine drinkers. Majority of people in rural China consumes rice wine rather than grape wine, so we combined them together to form a rice wine consumption group. Participants who consumed any kinds of alcoholic beverage at least 12 times last year were defined as a drinker in current study. All drinkers were classified into four categories based on their alcohol intake from, for example, all sources of alcoholic intake: non-drinkers, light drinkers (≤5.7 grams/day for women and ≤16.4 grams/day for men), moderate drinkers (≤17.7 grams/day for women and ≤45.2 grams/day for men), and severe drinkers (>17.7 grams/day for women and >45.2 grams/day for men). We investigated the intake amount of some foods in liang (=50 grams), including red meat (e.g., pork, beef, and lamb), white meat (e.g., chicken, duck, and goose), and fish. The data of other foods consumed, such as vegetables, fruits, and soy foods were also collected. We asked the participants how frequently (daily, weekly, monthly, yearly, or never) they consumed these food groups over the past year, followed by a question on the amount consumed in liang per unit of time. Exercise was defined as the participants who performed physical activities, such as Qigong, jogging, and basketball, during their leisure time. Tea consumption was defined as drinking tea more than two times per week for at least six months continuously. Socio-demographic factors, such as age at interview, education (none, elementary school, middle/high school, college, and above), personal income in Yuan/month (≤500, 501–1000, ≥1001) and occupation, were analyzed in the study as potential confounders.

### Anthropometric and biochemical measurements

According to standard protocol, anthropometric measurements of weight, height and waist circumference were taken twice for each participant during the in-person interviewers, which might help to prevent reading and typing errors. A third measurement was taken if difference between the first two measurements was larger than 1 cm for height and waist circumference or 1 kilogram (kg) for weight. The intra-observer variations of the three anthropometric measures were small, and the average coefficients of variation were 0.27%, 0.02%, and 0.24% for height, weight and waist circumference, respectively. Therefore, the average of two readings of height, weight, and waist circumference was used in our study. Based on the anthropometric measures, body mass index (BMI) was calculated as weight in kilograms divided by the square of height in meters.

Systolic blood pressure (SBP) and diastolic blood pressure (DBP) were measured three times with a standardized mercury sphygmomanometer after the participants had rested for five minutes or longer in a sitting position. The mean of the three measurements was used in the analysis.

To measure serum lipids and glucose, a 10 mL-overnight fasting blood sample was drawn into an EDTA vacutainer tube at the time of the in-person interview. The fasting time was verified before the blood sample collection, and the participants who had not fasted for at least eight hours did not have their blood drawn. The samples were stored in a portable Styrofoam box with ice packs (0-4°C) and were sent to a central CDC laboratory in Nantong. Serum samples were obtained by centrifugation. Glucose and lipid levels were measured within six hours of the sample separation. We stored the remaining specimens at −70°C to conduct other laboratory assays. Serum glucose, HDL cholesterol and triglyceride levels were analyzed enzymatically using reagents from the Shino-Test Corporation, Japan. An automated chemistry analyzer (Hitachi 7180, Tokyo, Japan) was used to measure the serum levels of glucose and the lipid profiles of the 20,502 participants in the Nantong CDC. Both inter- and intra- assay variations (coefficient of variation, CV) were less than 3.5% for glucose, TG, and HDL-c.

### Criteria for the MS diagnosis

Criteria of metabolic syndrome diagnosis according to a joint interim statement of the International Diabetes Federation Task Force on Epidemiology and Prevention; National Heart, Lung, and Blood Institute; American Heart Association; World Heart Federation; International Atherosclerosis Society; and International Association for the Study of Obesity [27] by adopting the Asian criteria for waist circumference. MS was defined as the presence of any 3 or more of the following 5 metabolic risk factors: (1) central obesity (waist circumference ≥80 cm for Chinese women and ≥85 cm for Chinese men); (2) elevated TG (fasting serum TG ≥ 1.7 mmol/L or taking abnormal lipid medication); (3) reduced HDL-c (fasting serum HDL-c <1.3 mmol/L for Chinese women and HDL-c <1.0 mmol/L for Chinese men or specific treatment for this lipid abnormality); (4) elevated blood pressure (SBP ≥130 mmHg or DBP ≥85 mmHg or taking hypertension medication); (5) elevated fasting glucose (serum glucose level ≥6.0 mmol/L or taking diabetes medication).

### Statistical analyses

Demographic, dietary and lifestyle characteristics were described as the mean ± standard deviation (SD) (or Median ± IQR) for continuous variables and percentages for categorical variables. These parameters were compared between the drinkers and non-drinkers using ANOVA for continuous variables with normal distribution, Wilcoxon rank sum test for continuous variables with non-normal distribution, and chis-square test for categorical variables. ANOVA was also applied to compare MS components between any type of alcoholic beverages drinkers and non-drinkers. Odds Ratios (ORs) and 95% confidence intervals (CIs) were estimated using non-conditional logistic regression to assess the associations between alcohol consumption and MS components. The regression model was adjusted for age at interview, BMI, education, marriage status, personal income, occupation, exercise, smoking status, tea consumption, and the intake of meat, fish, soy food, fruits and vegetables. The test for linear trend was performed by entering the ordinal exposure (e.g., the amount of alcohol from beer, liquor, rice wine and all sources) as continuous parameters in the models. A restricted cubic spline model was used for curve fitting between the ORs of MS components (TG, HDL-c, glucose, SBP and DBP, waist circumference) and alcohol consumption (continuous variable), and the figures present the curve fitting results. All p values presented were based on two-tailed test, and p < 0.05 was considered statistically significant. All analyses were performed using SAS statistical software (version 9.2; SAS Institute, Cary, NC).

## Results

### Characteristics of study participants

Differences of selected demographic characteristics, lifestyle factors, anthropometric measurements and some food intakes between drinkers and non-drinkers are reported in Table 1. There were 5,253 (25.6%) drinkers including 1,506 (11.2%) female and 3,747 (53.6%) male in our study. The MS prevalence was 20.6% for drinkers, which was 3.0% lower than that for non-drinkers (P = 0.009) in women. But the prevalence of MS was comparable between drinkers and non-drinkers in men. Drinkers were more likely to be smokers and tea consumers, to have healthy vegetables among all participants. Female drinkers were older and most of them were farmers. They were less educated and less likely to be obesity and to take fruit and soy food, compared with female non-drinkers. But male drinkers were younger and had higher income, more likely to be obesity and to take meat and less likely to be farmers.

**Table 1:** **Characteristics of the study population by gender and drinking status** *

	**All participants**	**Women**		**p values**	**Men**		**p values**
		**Drinker (n = 1,506)**	**Non-drinker (n = 11,999)**		**Drinker (n = 3747)**	**Non-drinker (n = 3250)**	
Age at interview (yeas, median ± IQR^†^)	55.7 ± 18.2	57.8 ± 14.2	53.8 ± 17.7	<0.001	57.6 ± 15.3	59.5 ± 16.9	<0.001
Weight (kg, mean ± SD)	60.2 ± 0.1	58.1 ± 0.3	58.2 ± 0.1	0.855	64.7 ± 0.2	63.6 ± 0.2	<0.001
BMI (kg/m^2^, mean ± SD)	23.7 ± 0.0	23.7 ± 0.1	24.0 ± 0.0	0.029	23.3 ± 0.1	23.2 ± 0.1	0.020
Red meat (g/day, median ± IQR)	18.9 ± 22.8	18.9 ± 24.2	18.9 ± 24.2	0.169	25.1 ± 25.1	18.9 ± 18.9	<0.001
White meat (g/day, median ± IQR)	10.8 ± 18.2	10.8 ± 17.0	10.8 ± 17.2	0.473	14.1 ± 18.2	10.8 ± 18.2	<0.001
Fish (g/day, median ± IQR)	22.1 ± 34.1	20.4 ± 34.1	20.4 ± 26.6	0.159	22.1 ± 31.0	22.1 ± 35.4	<0.001
Vegetables (g/day, median ± IQR)	225.0 ± 187.5	267.9 ± 187.5	225.0 ± 225.0	0.013	300.0 ± 187.5	262.5 ± 225.0	0.017
Fruits (g/day, median ± IQR)	17.5 ± 41.5	13.7 ± 41.5	17.5 ± 41.5	0.003	18.0 ± 41.3	18.3 ± 41.3	0.626
Soy food (g/day, median ± IQR)	28.6 ± 57.1	28.0 ± 35.7	28.6 ± 57.1	<0.001	32.9 ± 57.1	32.9 ± 57.1	0.450
MS (%)	21.1	20.6	23.6	0.009	16.4	17.1	0.477
Education (%)							
Primary school/under	59.7	69.1	63.3		50.8	52.8	
Middle school	27.9	22.0	26.9		31.1	30.1	
High school/above	12.4	8.9	9.8	<0.001	18.1	17.1	0.057
Marriage (%)							
Yes	90.0	90.7	90.1		91.1	88.2	
No^#^	10.0	9.3	9.9	0.644	9.0	11.8	<0.001
Income per person (%)							
≤500 Yuan	64.3	63.0	65.2		61.4	64.4	
501-1000 Yuan	29.1	29.6	29.2		29.8	28.0	
≥1001 Yuan	6.6	7.4	5.6	0.294	8.8	7.7	0.007
Farmer (%)							
Yes	64.9	73.5	68.6		55.3	59.0	
No	35.1	26.5	31.4	<0.001	44.7	41.0	<0.001
Ever smoked (%)							
Yes	18.8	11.5	2.7		58.3	35.4	
No	81.2	88.5	97.3	<0.001	41.7	64.6	<0.001
Tea consumption (%)							
Yes	15.2	15.1	10.0		28.4	19.3	
No	84.8	84.9	90.0	<0.001	71.6	80.7	<0.001
Exercise (%)							
Yes	9.3	8.8	10.1		8.2	7.7	
No	90.7	91.2	89.9	0.092	91.8	92.3	0.508

*Means, percentages and their p values were adjusted for age at interview. ^†^IQR: inter-quartile range (25-75th percentiles). ^#^Including widowed, divorced/separated and single.

### Association of alcohol consumption and MS components

We found different levels of MS components between drinking status and three types of alcoholic beverages after adjusted for age at interview in Table 2. In general, drinkers, no matter what kind of alcoholic beverage they drunk, had a higher level of HDL-c, compared with non-drinkers. Female drinkers had a lower level of TG (P < 0.01), and SBP (P < 0.05), compared with non-drinkers. Furthermore, lower SBP were found among female participants who consumed beer, liquor or rice wine. But only lower level of TG was found in rice wine drinkers, compared with non-drinkers. There was no difference of waist circumference, glucose level, and DBP between female drinkers and non-drinkers. Differently, higher levels of waist circumference, TG, SBP, and DBP (all P < 0.01) were found in male drinkers, compared with non-drinkers. Also, higher levels of waist circumference, TG and DBP were detected in beer or liquor drinkers. While higher glucose and SBP were only found in liquor drinkers, compared with non-drinkers.

**Table 2:** **Comparison of the levels of MS components according to drinking status and the three types of alcoholic beverages** * **(mean ± SD)**

	**Drinker**			**Beer drinker**			**Liquor drinker**			**Rice wine drinker**		
	**Yes**	**No**	**p**	**Yes**	**No**	**p**	**Yes**	**No**	**p**	**Yes**	**No**	**p**
	**For women**											
Waist (cm)	81.0 ± 0.26	80.9 ± 0.09	0.88	81.4 ± 0.31	80.9 ± 0.09	0.13	81.5 ± 0.48	80.9 ± 0.09	0.22	80.8 ± 0.33	81.0 ± 0.09	0.58
TG (mmol/L)	1.35 ± 0.03	1.46 ± 0.01	<0.01	1.39 ± 0.04	1.46 ± 0.01	0.07	1.44 ± 0.06	1.45 ± 0.01	0.92	1.36 ± 0.04	1.46 ± 0.01	0.02
HDL-c (mmol/L)	1.66 ± 0.01	1.56 ± 0.01	<0.01	1.66 ± 0.01	1.56 ± 0.01	<0.01	1.65 ± 0.02	1.57 ± 0.01	<0.01	1.66 ± 0.01	1.56 ± 0.01	<0.01
Glucose (mmol/L)	4.48 ± 0.04	4.54 ± 0.01	0.10	4.51 ± 0.04	4.54 ± 0.01	0.57	4.48 ± 0.07	4.54 ± 0.01	0.39	4.45 ± 0.05	4.54 ± 0.01	0.06
SBP (mmHg)	120.3 ± 0.46	121.4 ± 0.16	0.02	120.3 ± 0.54	121.4 ± 0.16	0.05	119.6 ± 0.84	121.3 ± 0.15	0.05	119.6 ± 0.56	121.4 ± 0.16	<0.01
DBP (mmHg)	73.3 ± 0.28	73.5 ± 0.10	0.63	73.5 ± 0.33	73.4 ± 0.10	0.84	73.3 ± 0.51	73.5 ± 0.09	0.81	72.9 ± 0.34	73.5 ± 0.09	0.12
	**For men**											
Waist (cm)	83.3 ± 0.17	82.1 ± 0.18	<0.01	83.5 ± 0.20	82.3 ± 0.16	<0.01	84.1 ± 0.24	82.3 ± 0.14	<0.01	82.9 ± 0.21	82.7 ± 0.15	0.58
TG (mmol/L)	1.49 ± 0.02	1.35 ± 0.03	<0.01	1.50 ± 0.03	1.38 ± 0.02	<0.01	1.58 ± 0.03	1.37 ± 0.02	<0.01	1.45 ± 0.03	1.41 ± 0.02	0.35
HDL-c (mmol/L)	1.62 ± 0.01	1.48 ± 0.01	<0.01	1.62 ± 0.01	1.52 ± 0.01	<0.01	1.63 ± 0.01	1.53 ± 0.01	<0.01	1.64 ± 0.01	1.51 ± 0.01	<0.01
Glucose (mmol/L)	4.51 ± 0.03	4.45 ± 0.03	0.13	4.51 ± 0.03	4.47 ± 0.03	0.29	4.59 ± 0.04	4.45 ± 0.02	<0.01	4.46 ± 0.03	4.50 ± 0.02	0.40
SBP (mmHg)	126.2 ± 0.28	124.3 ± 0.30	<0.01	125.6 ± 0.34	125.2 ± 0.26	0.29	126.9 ± 0.40	124.8 ± 0.24	<0.01	125.5 ± 0.34	125.3 ± 0.26	0.63
DBP (mmHg)	76.4 ± 0.18	74.8 ± 0.19	<0.01	76.1 ± 0.21	75.3 ± 0.17	<0.01	77.1 ± 0.25	75.1 ± 0.15	<0.01	76.0 ± 0.22	75.4 ± 0.16	0.02

*Adjusted for age at interview.

Table 3 shows the association of MS components with consumption of several alcoholic beverages adjusted for age at interview, BMI, education, marriage status, personal income, occupation, exercise, smoking status, tea consumption and intake of meat, fish, soy food, fruits and vegetables among female participants. We found that rice wine consumption was associated with decreasing risk of high TG (OR = 0.88, 95% CI: 0.81-0.95), risk of high glucose (OR = 0.79, 95% CI: 0.67-0.93) and risk of low HDL-c (OR = 0.84, 95% CI: 0.77-0.91). There was no association between rice wine consumption and blood pressure or waist circumference. However, beer consumption was only negatively associated with high risk of TG (OR = 0.87, 95% CI: 0.80-0.95) and risk of low HDL-c (OR = 0.82, 95% CI: 0.75-0.90).

**Table 3:** **Associations of MS components with consumption of several alcoholic beverages among cases of high MS components and healthy women** *

	**TG**		**HDL**		**Glucose**		**SBP**		**DBP**		**Waist circumference**	
	**Cases (%)**	**OR (95% CI)**	**Cases (%)**	**OR (95% CI)**	**Cases (%)**	**OR (95% CI)**	**Cases (%)**	**OR (95% CI)**	**Cases (%)**	**OR (95% CI)**	**Cases (%)**	**OR (95% CI)**
Alcohol from beer (g/d)												
0	3078(26.6)	1.0	3048(27.4)	1.0	610(6.1)	1.0	3414(29.9)	1.0	1757(16.6)	1.0	6499(53.0)	1.0
≤3.1	102(24.5)	0.78(0.61-1.00)	83(21.0)	0.69(0.54-0.89)	14(3.9)	0.57(0.33-0.98)	123(29.9)	0.86(0.68-1.09)	71(18.3)	1.04(0.79-1.38)	257(59.0)	1.12(0.85-1.48)
≤6.2	86(23.4)	0.76(0.58-0.99)	73(20.4)	0.70(0.53-0.91)	22(6.7)	0.94(0.59-1.48)	121(32.3)	0.99(0.78-1.25)	64(18.5)	1.16(0.86-1.55)	217(54.5)	1.00(0.74-1.34)
>6.2	42(22.1)	0.72(0.50-1.04)	36(18.9)	0.61(0.42-0.88)	7(4.1)	0.56(0.26-1.22)	56(29.6)	0.81(0.57-1.15)	28(15.6)	0.85(0.55-1.32)	117(57.6)	1.30(0.88-1.92)
p for trend		0.0018		<0.0001		0.0723		0.2106		0.8447		0.2457
Alcohol from liquor (g/d)												
0	3206(26.4)	1.0	3158(27.0)	1.0	632(6.0)	1.0	3592(29.9)	1.0	1866(16.8)	1.0	6859(53.2)	1.0
≤6.2	41(24.6)	0.75(0.52-1.10)	39(24.8)	0.87(0.60-1.28)	11(7.6)	1.22(0.64-2.31)	55(33.3)	1.05(0.73-1.50)	25(17.1)	1.00(0.63-1.58)	95(54.0)	0.73(0.47-1.13)
≤20.5	26(24.8)	0.71(0.44-1.14)	17(17.4)	0.53(0.31-0.92)	2(2.3)	0.29(0.07-1.18)	25(25.8)	0.64(0.39-1.04)	9(9.7)	0.47(0.23-0.95)	62(57.9)	0.91(0.50-1.64)
>20.5	38(28.2)	1.00(0.66-1.49)	27(20.5)	0.70(0.45-1.09)	10(8.3)	1.11(0.56-2.20)	44(32.4)	0.88(0.59-1.31)	20(16.3)	0.90(0.54-1.51)	82(56.2)	1.15(0.71-1.87)
p for trend		0.2644		0.0105		0.6687		0.1981		0.1830		0.9317
Alcohol from rice wine (g/d)												
0	3093(26.7)	1.0	3070(27.4)	1.0	618(6.1)	1.0	3419(29.8)	1.0	1788(16.8)	1.0	6561(53.1)	1.0
≤4.1	68(22.4)	0.65(0.49-0.87)	60(20.6)	0.66(0.49-0.88)	12(4.5)	0.61(0.33-1.10)	90(29.7)	0.82(0.63-1.08)	42(15.1)	0.79(0.55-1.12)	183(56.8)	0.94(0.68-1.30)
≤12.8	73(24.6)	0.74(0.56-0.99)	50(17.9)	0.60(0.43-0.82)	12(4.6)	0.57(0.31-0.98)	91(31.5)	0.79(0.60-1.04)	46(17.0)	0.96(0.68-1.35)	174(57.1)	1.05(0.75-1.46)
>12.8	78(23.7)	0.76(0.58-0.98)	63(19.8)	0.70(0.53-0.94)	13(4.5)	0.57(0.32-0.99)	117(36.7)	1.10(0.85-1.42)	43(14.8)	0.87(0.62-1.23)	181(53.9)	1.17(0.85-1.61)
p for trend		0.0013		<0.0001		0.0044		0.5820		0.2929		0.4059
All sources of alcohol (g/d)												
0	2982(26.8)	1.0	2972(27.7)	1.0	593(6.2)	1.0	3265(29.7)	1.0	1701(16.7)	1.0	6262(52.9)	1.0
≤5.7	109(23.4)	0.72(0.57-0.91)	87(19.7)	0.62(0.49-0.80)	16(4.0)	0.52(0.31-0.87)	138(29.9)	0.85(0.68-1.07)	81(18.5)	1.06(0.81-1.38)	278(56.1)	0.99(0.76-1.29)
≤17.7	111(23.6)	0.72(0.57-0.90)	105(22.9)	0.78(0.62-0.98)	18(4.4)	0.56(0.35-0.92)	151(32.1)	0.90(0.73-1.12)	66(15.4)	0.84(0.64-1.21)	285(57.9)	1.20(0.92-1.55)
>17.7	110(23.4)	0.73(0.57-0.92)	79(17.4)	0.57(0.44-0.74)	27(6.4)	0.81(0.53-1.23)	162(35.1)	1.00(0.81-1.25)	72(16.7)	0.99(0.75-1.30)	274(55.7)	1.19(0.91-1.55)
p for trend		<0.0001		<0.0001		0.0144		0.4517		0.5634		0.1018

*Abnormal TG, HDL, Glucose and blood pressure, respectively. All models were adjusted for age at interview, BMI, education, marriage status, personal income, occupation, exercise, smoking status, tea consumption, and intake of meat, fish, soy food, fruits and vegetables.

The associations of MS components with consumption of several alcoholic beverages among male participants are presented in Table 4. Similar to that among women, a decreased risk of having low HDL-c was found in rice wine (OR = 0.75, 95% CI: 0.66-0.87), beer (OR = 0.74, 95% CI: 0.65-0.85), and liquor drinkers (OR = 0.82, 95% CI: 0.71-0.94), compared with non-drinkers. Moreover, liquor consumption was associated with increasing risk of high SBP (OR = 1.08, 95% CI: 1.03-1.15), high DBP (OR = 1.15, 95% CI: 1.08-1.23), and high waist circumference (OR = 1.11, 95% CI: 1.02-1.20). There was no association between liquor consumption and TG or glucose level.

**Table 4:** **Associations of MS components with consumption of several alcoholic beverages among cases of high MS components and healthy men** *

	**TG**		**HDL**		**Glucose**		**SBP**		**DBP**		**Waist circumference**	
	**Cases (%)**	**OR (95% CI)**	**Cases (%)**	**OR (95% CI)**	**Cases (%)**	**OR (95% CI)**	**Cases (%)**	**OR (95% CI)**	**Cases (%)**	**OR (95% CI)**	**Cases (%)**	**OR (95% CI)**
Alcohol from beer (g/d)												
0	873(21.2)	1.0	241(6.4)	1.0	222(5.9)	1.0	1457(34.9)	1.0	804(20.3)	1.0	1668(38.8)	1.0
≤6.2	314(22.6)	0.94(0.79-1.10)	48(3.8)	0.49(0.35-0.68)	70(5.4)	0.81(0.61-1.09)	485(34.3)	1.00(0.87-1.14)	273(20.0)	0.92(0.78-1.09)	630(43.5)	1.15(0.95-1.39)
≤12.3	71(27.7)	1.11(0.80-1.52)	9(3.9)	0.41(0.20-0.84)	10(4.4)	0.66(0.34-1.29)	93(35.8)	1.08(0.82-1.43)	53(21.8)	1.00(0.71-1.39)	127(46.9)	1.29(0.89-1.87)
>12.3	230(27.4)	1.15(0.95-1.39)	32(4.3)	0.51(0.34-0.75)	34(4.5)	0.70(0.47-1.03)	290(34.4)	1.07(0.91-1.27)	177(21.8)	1.03(0.84-1.26)	374(43.1)	1.07(0.85-1.36)
p for trend		0.1967		<0.0001		0.0308		0.3972		0.9232		0.2986
Alcohol from liquor (g/d)												
0	1049(21.4)	1.0	258(5.8)	1.0	240(5.4)	1.0	1675(33.7)	1.0	893(18.9)	1.0	1982(38.8)	1.0
≤12.3	130(25.0)	1.01(0.80-1.28)	21(4.5)	0.60(0.37-0.97)	30(6.3)	0.97(0.64-1.47)	174(33.7)	0.99(0.80-1.22)	103(20.6)	1.03(0.81-1.32)	245(45.4)	1.32(0.99-1.76)
≤37.0	180(26.6)	1.16(0.94-1.43)	32(5.2)	0.72(0.48-1.07)	35(5.7)	0.96(0.66-1.41)	256(36.9)	1.14(0.95-1.36)	164(24.7)	1.32(1.08-1.63)	318(44.9)	1.20(0.93-1.54)
>37.0	136(25.9)	1.03(0.82-1.31)	19(4.1)	0.55(0.33-0.90)	33(6.9)	1.07(0.71-1.59)	228(42.6)	1.32(1.08-1.60)	150(29.0)	1.55(1.24-1.94)	259(47.4)	1.34(1.00-1.78)
p for trend		0.3567		0.0033		0.8943		0.0049		<0.0001		0.0147
Alcohol from rice wine (g/d)												
0	966(22.8)	1.0	251(6.5)	1.0	229(5.9)	1.0	1464(34.0)	1.0	828(20.3)	1.0	1793(40.4)	1.0
≤9.0	182(22.7)	0.98(0.80-1.20)	34(4.6)	0.66(0.45-0.96)	38(5.2)	0.78(0.54-1.12)	269(33.6)	0.94(0.79-1.12)	148(19.3)	0.91(0.74-1.12)	348(42.0)	1.17(0.92-1.48)
≤25.7	213(21.7)	1.02(0.85-1.23)	28(3.1)	0.49(0.33-0.74)	48(5.3)	0.86(0.61-1.20)	358(35.8)	1.07(0.92-1.25)	208(21.5)	1.11(0.92-1.33)	422(41.5)	1.28(1.03-1.59)
>25.7	136(22.4)	1.09(0.86-1.37)	18(3.3)	0.54(0.32-0.89)	23(4.2)	0.67(0.43-1.06)	244(39.3)	1.24(1.03-1.50)	127(21.6)	1.14(0.91-1.43)	244(39.1)	1.01(0.77-1.32)
p for trend		0.5419		<0.0001		0.0587		0.0400		0.1856		0.1770
All sources of alcohol (g/d)												
0	652(21.2)	1.0	198(7.0)	1.0	162(5.8)	1.0	1005(32.4)	1.0	550(18.7)	1.0	1242(38.6)	1.0
≤16.4	283(24.0)	1.05(0.88-1.25)	54(5.1)	0.61(0.44-0.85)	67(6.3)	1.02(0.75-1.38)	417(35.1)	1.14(0.98-1.33)	218(19.5)	1.00(0.83-1.21)	506(41.5)	0.95(0.76-1.17)
≤45.2	259(21.7)	0.94(0.78-1.13)	43(3.9)	0.46(0.32-0.65)	49(4.5)	0.66(0.47-0.94)	436(35.9)	1.19(1.02-1.38)	258(22.0)	1.18(0.99-1.41)	537(43.1)	1.32(1.07-1.64)
>45.2	300(25.3)	1.16(0.97-1.39)	36(3.4)	0.40(0.27-0.58)	60(5.6)	0.86(0.62-1.19)	474(39.2)	1.33(1.14-1.55)	284(24.5)	1.37(1.14-1.63)	521(42.6)	1.14(0.92-1.42)
p for trend		0.2925		<0.0001		0.0926		0.0002		0.0005		0.0517

*Abnormal TG, HDL, Glucose and blood pressure, respectively. All models were adjusted for age at interview, BMI, education, marriage status, personal income, occupation, exercise, smoking status, tea consumption, and intake of meat, fish, soy food, fruits and vegetables.

**Figure 1:**
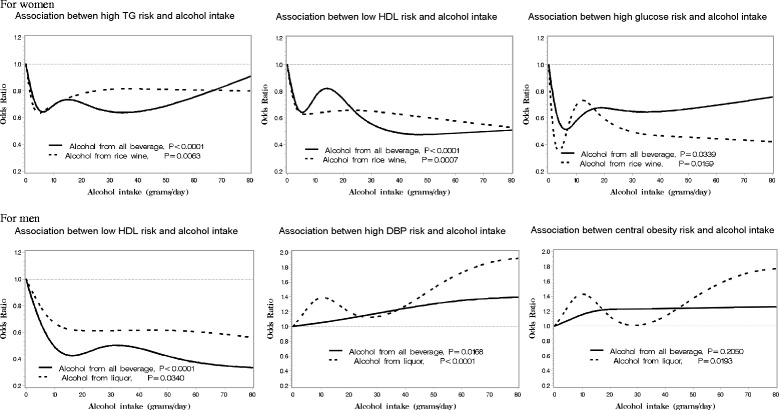
**Association between alcohol consumption and MS components risk.** Legend: Association between the MS components risk and various alcohol beverage intakes in 13,505 women and 6997 men in rural China; the NMSS during 2007–2008 and all p value were from linear trend test.

Associations of all alcoholic beverage and rice wine in women or liquor in men with MS components are depicted in Figure 1. In women we found that both all alcoholic beverage and rice wine had strong protective effect on high TG, high blood glucose and low HDL when consumption of alcohol was less than 10 grams per day. The protective effect of rice wine was increased a little bit on high glucose and low HDL if rice wine consumption was more than 10 grams per day. In contrast, both all alcoholic beverage and liquor were significantly associated with the prevalence of high BP in men. Also, a similar association was found between liquor and the prevalence of central obesity.

## Discussion

In this cross-sectional study among adults in rural China, alcohol consumption was associated with a lower prevalence of MS in women and any alcoholic beverage might decrease the risk of low HDL-c in both men and women. In particular, we observed that both rice wine and light to moderate alcoholic beverage consumption had a significantly protective effect on high TG, and high glucose with a non-linear relationship among women, but not among men. However, severe liquor and alcoholic beverage consumption was found to increase risk of high BP and central obesity among men only.

Reports on the relationship between alcohol consumption and MS were inconsistent. Some studies have shown a positive, linear relationship between alcohol consumption and MS [1,28,29] and this association was significantly higher in men aged 50 years or older, whereas no significant association was observed in women [30]. Other studies reported that mild-to-moderate alcohol consumption decreased the risk of the MS in all participants [31] or in women only [32]. In our previous study, we found that rice wine consumption may decrease the risk of the MS in women and no associations were found in beer or liquor consumption [25]. These inconsistent results might be related to the different type and amount of alcoholic beverage consumption, the complex mechanistic relationship between alcohol consumption and each component of MS, and some confounding factors such as race and sex [28,29,33].

One of favorable effects of alcohol consumption is to decrease the risk of having low HDL-c levels [34]. We found that a dose–response relation between HDL-c levels and the quantity of beer, rice wine, liquor or combined alcoholic beverage consumption in both male and female participants. This result is consistent with early studies [7,9,10,18,22,35]. A linear association between alcohol and wine intake with the number of HDL particles has been documented [36]. However, we found the association was stronger among rice wine or beer drinkers than that of combined alcoholic beverage drinkers, suggesting that additional components polyphenols in wine other than alcohol might play an important role on lipid profiles, through its anti-inflammatory, insulin-sensitizing and vasodilatory properties [37]. It is possible that alcohol consumption (especially rice wine and beer) increased the hepatic production of apolipoproteins and TG lipase concentrations and decreased the removal of circulating HDL-c [38,39].

In current study, we found rice wine consumption was related with constantly decreasing risk of high TG and high glucose in female drinkers, and also the risk of both high TG and high glucose was decreased rapidly if a woman drank all alcoholic beverage less than 10 g/d and this protective effect was gradually leveled off if drinking more than 10 g/d. Our findings are consistent with that in previous studies in which they found light-to-moderate alcohol consumption may be associated with decreased triglycerides [19,40-42] and blood glucose [22,43]. The mechanism of this protective effect is plausible. Several studies suggested that the polyphenols enriched in red wine possess multiple benefits on MS and its components beyond alcohol through their anti-oxidant, anti-inflammatory, vascular-protective and insulin-sensitizing properties [22,37]. Resveratrol, a polyphenolic compound enriched in red wine, has recently attracted enormous attention because of combating the ageing process induced by nutrient excess [44]. Furthermore, Chinese rice wine contains large amount of polyphenol substance with strong ability of antioxidation [45], which is similar to that of red wine and may partially explain association of rice wine consumption and low risk of MS components among women.

Interestingly, protective effects in female drinkers were not found in male drinkers in current study. Conversely, we observed that alcoholic beverage may increase risk of high BP and central obesity among male drinkers, especially for liquor drinkers. This result is similar with several studies. In a meta-analysis, McFadden *et al*. reported that daily alcohol intake increased SBP by 2.7 mmHg and DPB by 1.4 mmHg [46]. Several prospective studies reported that no association of increasing risk of hypertension with light-to-moderate level of alcohol consumption in white men [15] but excessive alcohol consumption could lead to hypertension [16]. The difference of association of alcoholic beverage consumption and MS components between males and females might be due to unhealthy drinking patterns, including quantity and types of alcoholic beverage consumed [30,47]. Literature review showed high amount of alcohol intake remains harmful by elevating triglycerides [35,48]. Alcohol-induced hypertriglyceridemia, due to increased very-low-density lipoprotein secretion, impaired lipolysis and increased free fatty acid fluxes from adipose tissue to the liver. While light-to-moderate alcohol consumption may be associated with decreased triglycerides [19,40-42], reduce 30% of diabetes risk and the risk of having high blood glucose in male [22,43], and no association of increasing risk of hypertension in white men [15]. In agree with previous studies, male drinkers had 2–3 times of alcohol consumption and may lead to harmful effect of alcoholic beverage on MS components, compared with female drinkers in current study. Severe drinkers, no matter what type of alcoholic beverage consumed, may associate with obesity. We did not find association between any alcohol beverages and waist circumference in Chinese women because of their light-to-moderate alcohol consumption. It is consistent with previous study in Japan [49] and America [24]. But we found liquor was associated with waist circumference in men, which is in line with previous studies. Severe alcohol consumers (≥50.0 g/d) had an increased risk of central obesity in middle-aged and elderly Chinese [43], especially in men [50,51] and in beer or liquor drinkers [50] because beer or liquor provides more energy than other types of alcoholic beverages and severe drinkers may lead to obesity because of excessive energy intake, lipid oxidation and fat accumulation [52]. A recent study demonstrated that alcohol also inhibits ghrelin secretion, resulting in appetite stimulation among men [53]. Moreover, type of alcoholic beverage may play an impotent role in association with MS components. Kao *et al.* found that severe liquor drinking was shown to increase the risk of type II diabetes [54], whereas similar amount of beer or wine did not, implying this effect is related to types of alcoholic beverage. Given rice wine has protective effect on MS, high TG and high glucose, unhealthy drinking pattern, especially for sever drink, could attenuate this effect among male participants in our study.

The strengths of this study include using a large pool of population-based data, extensive information on confounders, and standard methods and vigorous quality control to measure all variables. The study does, however, have several limitations and considerations. First, given the cross-sectional design, we could not draw any causal inference regarding the association of alcohol consumption with MS components. Therefore, future prospective studies are required to confirm our findings. Second, alcohol consumption status was based on the results of self-reported questionnaires, which might have recall bias. Finally, and we cannot do more significant analysis by combining years and amount of drinking together because ‘years of drinking’ is not available.

## Conclusions

In summary, alcohol consumption is associated with a lower prevalence of MS among Chinese women. A favorable effect on TG and glucose is found among rice wine consumers in female and light-to-moderate combined alcoholic consumers. Any type of alcohol beverage, including beer, liquor, and rice wine might decrease the risk of lower HDL-c in both genders. But beneficial effect of alcoholic consumption was offsetting by severe drinkers in men, which lead to no association between alcohol consumption and MS in our study. Our data provide an evidence that rice wine and healthy drinking patterns have a beneficial effect on serum lipids and serum glucose, and has better protective effects than any other alcoholic beverages on MS components among Chinese women.

## Abbreviations

MS: Metabolic syndrome
NMSS: Nantong metabolic syndrome study
TG: Triglycerides
HDL-c: High-density lipoprtein cholesterol
BP: Blood pressure
SBP: Systolic blood pressure, DBP, Diastolic blood pressure
IDF: International diabetes federation
CDC: the Nantong centers for disease control
BMI: Body mass index
ORs: Odds ratios
CIs: Confidence intervals
